# Faecal egg count reduction test, deep amplicon sequencing of isotype-1 β-tubulin gene and *in ovo* larval development assay reveal susceptibility to benzimidazoles of porcine nematodes *Oesophagostomum* spp. and *Ascaris suum* in outdoor-reared pigs in Germany

**DOI:** 10.1016/j.ijpddr.2025.100612

**Published:** 2025-08-28

**Authors:** Hannah RM. Fischer, Jürgen Krücken, Stefan Fiedler, Stig M. Thamsborg, Hendrik Nienhoff, Stephan Steuber, Ricarda Daher, Georg von Samson-Himmelstjerna

**Affiliations:** a Institute for Parasitology and Tropical Veterinary Medicine, Freie Universität Berlin, Robert-von-Ostertag-Str. 7, 14163, Berlin, Germany; b Veterinary Centre for Resistance Research (TZR), Freie Universität Berlin, Robert-von-Ostertag-Str. 8, 14163, Berlin, Germany; c Federal of Consumer Protection and Food Safety, Berlin, Germany; d Department of Veterinary and Animal Sciences, University of Copenhagen, Frederiksberg, Denmark; e Institute for Animal Health (Lufa-Nord-West), Ammerländer Heerstr. 123, 26129 Oldenburg, Germany

**Keywords:** Anthelmintic resistance, FECRT, LDA, Beta-tubulin, Next generation sequencing

## Abstract

*Oesophagostomum* spp. and *Ascaris suum* represent the most common porcine nematodes and anthelmintic resistance (AR) to various anthelmintics has been reported for *Oesophagostomum*. However, the current AR status for worm populations on German farms and practical methods facilitating reliable AR detection are missing. Herein, the efficacy of benzimidazoles (BZ) (fenbendazole, 5 mg/kg body weight, single dose) was analysed on 13 farms with outdoor access. The Faecal Egg Count Reduction Test (FECRT) estimates for strongyles on the farms (range 99.8–100 %) exceeded the target efficacy (99 %) of the new W.A.A.V.P. guideline for *Oesophagostomum dentatum.* Deep amplicon sequencing was used for the first time for porcine nematodes and revealed no polymorphisms associated with BZ-resistance in codons 134, 167, 198 and 200 of the isotype-1 β-tubulin gene. Nemabiome analysis using ITS-2 deep amplicon sequencing, based on two pre- and post-treatment samples, showed a significant increase (p < 0.001) of *Oesophagostomum quadrispinulatum* after BZ treatment. For *A. suum,* the interpretation of FECRT estimates can be hindered due to coprophagy-associated false-positive egg counts in pigs. Therefore, two FECRT analysis for *A. suum* were pursued, the first analyses included all EPG data, the second considered EPGs <200 pre- and post-treatment as negative. An *in ovo* larval development assay (LDA) was developed for the *in vitro* analysis of BZ-susceptibility in *A. suum*. Computed EC_50_ values ranged from 1.50 to 3.36 μM thiabendazole (mean 2.24 μM). An EC_50_ of 3.90 μM thiabendazole (mean EC_50_ + 3 × SD) as provisional cut-off for detection of resistant populations is suggested. In conclusion, no AR was detected in *Oesophagostomum* using the FECRT and β-tubulin deep amplicon sequencing. For *A. suum* the FECRT results were ambiguous*,* in some cases even when excluding the low egg counts from calculations. With the *in ovo* LDA all investigated *A. suum* populations were identified as susceptible to BZ.

## Graphical abstract

**Figure:**
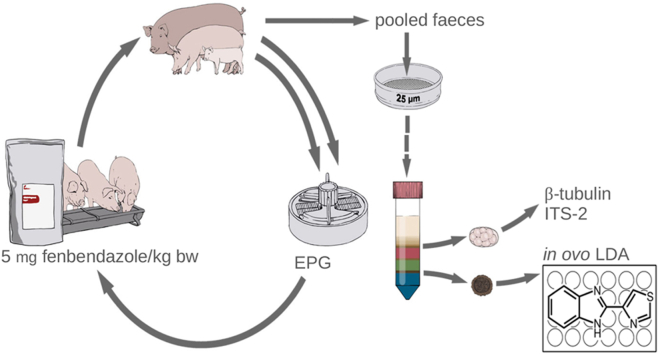

## 1 Introduction

Species of the nodular worms in the genus *Oesophagostomum* and the large roundworm *Ascaris suum* are widely distributed around the world and can be found in conventional and alternative pig farming systems (Eijck and Borgsteede, 2005; Haugegaard, 2010; Kochanowski et al., 2017; Lindgren et al., 2020; Pettersson et al., 2021b; Hernandez et al., 2023). However, farms with alternative farming systems, i.e. organic farms or other farms with outdoor pens or pasture access (in the following text, the term ‘alternative’ is used for pigs or farms with outdoor or pasture access), are associated with higher prevalences than intensive indoor systems (Eijck and Borgsteede, 2005; Kochanowski et al., 2017).

Porcine gastrointestinal nematode infections tend to cause subclinical disease (Roepstorff et al., 2011; Thamsborg et al., 2013). Nevertheless, a high worm burden is associated with a decrease in performance, welfare problems, individuals may develop clinical disease (Pattison et al., 1979, 1980; Forsum et al., 1981; Hale et al., 1981, 1985; Knecht et al., 2011, 2012; Jankowska-Mąkosa and Knecht, 2015; Whitehead et al., 2022) and infections with either species may have a negative economic impact. Additionally, pigs have been confirmed as a source of *Ascaris* infections in humans in particular in regions that are non-endemic for human ascariosis (Anderson, 1995; Nejsum et al., 2005; Dold and Holland, 2011; Cavallero et al., 2013). One single female of *A. suum* has been estimated to produce more than 1 million eggs per day (Kelley and Smith, 1956; Olsen et al., 1958). Thus, using manure as fertiliser or water contaminated with pig faeces can quickly lead to the contamination and transmission of ascarid eggs onto products intended for human consumption (Nejsum et al., 2005; Bowman, 2021). Two reports on food-born infections with *A. suum* in children in Denmark (Roepstorff et al., 2011) and a report on an *Ascaris* infection outbreak from imported vegetables in Finland (Räisänen et al., 1985) have been published previously.

In Germany, benzimidazole (BZ) and macrocyclic lactone (ML) based products represent the majority of the authorised veterinary anthelmintic products for use in pigs (Federal Institute for Drugs and Medical Devices, 2025). At the time of writing, 13 BZ products with the active components flubendazole or fenbendazole (FBZ), 16 ML products (doramectin and ivermectin), and 6 levamisole products were available on the market in Germany (Federal Institute for Drugs and Medical Devices, 2025). Accordingly, five different anthelmintic active compounds from only three different drug classes are currently available for worm control on German pig farms. According to EU Regulation 2018/848, the withdrawal period for parasite treatment on organic farms must be twice as long as the withdrawal periods in conventional herds. As a result, farmers will most likely favour treatments with BZs, as they usually have a considerably shorter withdrawal period (Federal Institute for Drugs and Medical Devices, 2025) than products containing MLs. In addition, some organic associations in Germany (e.g. Demeter, BIOLAND) have guidelines that go beyond EU regulations and prohibit the use of MLs to treat endoparasite infections (Bioland e.V., 2024; Demeter, 2025).

In the early 1960s the BZs were the first class of broad-spectrum anthelmintics introduced into the market and they are widely used since then (Krücken et al., 2012). Anthelmintic resistance (AR) is known to be rapidly selected in strongyle nematodes under drug pressure (Knapp-Lawitzke et al., 2015) and has become a wide spread problem in small ruminants, cattle and horses (Rose Vineer et al., 2020; Charlier et al., 2022; Nielsen, 2022) causing severe economic losses (Charlier et al., 2020, 2022). Until now, for porcine nematodes there are almost exclusively reports about AR in the nodular worms *Oesophagostomum* spp. The first report of AR in *Oesophagostomum* spp. described pyrantel resistance in Denmark (Roepstorff et al., 1987), followed by a report describing levamisole and pyrantel cross-resistance in Denmark (Bjørn et al., 1990). Levamisole and piperazine resistance in *Oesophagostomum* spp. were also detected in Kenya (Kagira et al., 2003). Levamisole and BZ resistance were found in *Oesophagostomum* spp. on three German farms (Gerwert et al., 2002) and more recent studies detected ivermectin resistance in England (Macrelli et al., 2019) and Sweden (Pettersson et al., 2021a). To date, there is only one report on AR in other porcine nematode species, in particular *Trichuris suis* resistance to levamisole (Kagira et al., 2003). However, this does not necessarily reflect the current situation in the field. As Kaplan et al. (2023) noted, considerably fewer investigations have been carried out on pig nematodes compared to other livestock species. To the best knowledge of the authors, no further studies investigating AR on German farms on either *Oesophagostomum* spp. or *A. suum* have been published. Consequently, there is a knowledge gap concerning the occurrence of AR in porcine nematodes in both conventional and alternative farming systems. Furthermore, reduced efficacy of BZs against ascarids in humans has been reported (Krücken et al., 2017). In addition to the economic losses linked to high prevalences of gastrointestinal nematode infections in pigs, the potential for human infections with resistant *A. suum* should be considered in the risk assessment of the emergence of porcine nematode resistance.

The most common approach, and currently the method of choice (Kaplan et al., 2023), for AR detection is the faecal egg count reduction test (FECRT), an *in vivo* testing based on faecal egg counts pre- and post-treatment. Regarding pigs, the recently revised guideline of the World Association for the Advancement of Veterinary Parasitology (W.A.A.V.P) provides detailed information on the implementation as well as the interpretation of the FECRT only for the nodular worm *O. dentatum* while it refers to difficulties of very high variance of *A. suum* egg counts and occasional observations of high post-treatment egg counts (Kaplan et al., 2023). Several other methods for AR detection in strongyles have been established such as the Egg Hatch Assay (EHA), the Larval Development Assay (LDA), the Larval Migration Inhibition Assay (LMIA) and the Larval Feeding Inhibition Assay (LFIA) (Johansen, 1989; Jackson and Coop, 2000; Taylor et al., 2002; Demeler et al., 2012). For ascarids there are only two previously published LDA protocols for *Parascaris* spp. and *Ascaridia galli* (Tydén et al., 2016; Tarbiat et al., 2017). Overall, bioassays can supply valuable information on the *in vitro* susceptibility of parasite populations or communities. However, both the FECRT and the bioassays are labour intensive and time consuming. Additionally, the need to visit farms twice and the performance of individual egg counts make the FECRT relatively expensive, especially if performed commercially. Therefore, a molecular approach detecting mutations associated with a BZ-resistant phenotype was introduced by Avramenko et al. (2019) using deep amplicon sequencing. The deep amplicon sequencing provides an accurate measure of BZ-resistance associated β-tubulin allele frequencies and is suitable to detect early stages of BZ-resistance in a given worm population (Avramenko et al., 2019). It was shown that BZ-resistance associated isotype-1 β-tubulin alleles can be detected at a very low level, down to a frequency of 0.1 % (Avramenko et al., 2019). Various single nucleotide polymorphisms (SNPs) in the codons 167, 198 and 200 of the isotype-1 β-tubulin gene are associated with BZ-resistance in nematode species of ruminants (Roos et al., 1990; Kwa et al., 1993, 1994, 1995; Njue and Prichard, 2003; Gilleard, 2006; Keegan et al., 2017; Baltrušis et al., 2020; Martínez-Valladares et al., 2020; Mohammedsalih et al., 2020) and have been shown to induce resistance in the model organism *Caenorhabditis elegans* (Hahnel et al., 2018; Dilks et al., 2020, 2021). Recently, a new BZ-resistance inducing polymorphism at codon 134 was discovered in dog hookworms of the species *Ancylostoma caninum* in the USA (Venkatesan et al., 2023). Established for ruminant strongyles, the deep amplicon sequencing of the isotype-1 β-tubulin gene has never been used to screen for BZ-resistance associated mutations at codons 134, 167, 198 and 200 in porcine *Oesophagostomum* spp.

The following results are part of a larger study with the overall goal to describe parasite communities and their distribution in German alternatively reared pigs, as well as the occurrence of BZ-resistance of the most common porcine nematodes – *Oesophagostomum* spp. and *A. suum*. Previously published results (Fischer et al., 2024) showed that *Oesophagostomum* spp., *A. suum*, *T. suis*, *Metastrongylus* spp. and to a much smaller extend even the in wild boars common hookworm *Globocephalus urosubulatus* are present on German alternative pig farms. The nemabiome analysis based on the ITS-2 gene region (Avramenko et al., 2015) also showed that the nodular worms *O. dentatum* and *O. quadrispinulatum* often appeared as mixed infections. Additionally, a mixed-effect regression analysis was performed using questionnaire data obtained from the participating farms in order to identify potential risk factors (Fischer et al., 2024). In the present part of the study the aim was to collect up-to-date data on the occurrence of BZ-resistance in German alternatively reared pigs and to introduce two new methods to diagnose AR in porcine nematodes, a next-generation sequencing approach for *Oesophagostomum* spp. and an *in vitro* bioassay (*in ovo* LDA) for *A. suum.* Furthermore, it was aimed to perform nemabiome analyses on samples taken post-treatment in order to identify changes in species composition caused by BZ treatment.

## 2 Materials and methods

### 2.1 Farms

#### 2.1.1 Farm selection

Farm selection was not random but was based on the personal interest of the farmers to participate and their willingness to support the sampling process twice. Only German farms with alternative farming systems, i.e. concrete outdoor pens or access to pasture for some animals were included in this study. It was required that animals had not been treated with anthelmintics for at least six weeks. Farm distribution and characteristics were published elsewhere (Fischer et al., 2024).

#### 2.1.2 Questionnaire data

Data of each participating farm was obtained using a questionnaire previously published (Fischer et al., 2024).

### 2.2 Sample collection and treatment

Initially two age groups were targeted: Piglets after weaning, about 10 weeks old, and fatteners at the age of 24–26 weeks and approximately 4 weeks prior to slaughter. At least 10 animals of each age group were sampled per farm using a convenience-based approach. Due to the in parallel encountered high strongyle worm egg shedding in sows, it was decided to include sows for FECRT calculations. Individual rectal samples were taken from all animals with an examination glove and immediately cooled in a transportable cooler (4–8 °C). All sows, but also a few of the piglets and fatteners had individual ear tag numbers and were easily re-identified at the second sampling. In order to identify the other animals carrying ear tags indicating only the farm identity two weeks after the first sampling, several methods were approached simultaneously. Firstly, each animal was marked with blue spray (Covertrus Inc.) with an individual number (1–30). Secondly, the individual number was written with permanent marker on the inner blank part of the ear tag. Finally, individual characteristics such as sex, colouring, side (right/left ear) and colour of the ear tag, abnormities, rotation direction of the tail etc. were noted for each animal.

The animals were treated with FBZ (“Fenbendatat 5 %, 50 mg/g”, LIVISTO c/o aniMedica GmbH, 5 mg/kg bodyweight orally). When possible, the weight of the animals was determined with scales provided by the farmers for dose calculations. If no scale was available, the weight was estimated based on breed, body condition and size of the animals. The dose was calculated for the whole group in a pen, also taking into account animals that were not sampled, based on the heaviest animal in order to prevent underdosing. The amount of FBZ per group was measured with a scale. The application was carried out by the farmers via the feed on day one or two after the first sample was taken. Following the guidelines of the Federal Ministry of Food and Agriculture (BMEL), it was requested that the FBZ would be administered in a daily ration of feed (BMEL, 2014). A second sample was collected from the same individuals 10–13 days post-treatment. The variability of the post-treatment sampling was due to the availability of the farmers and the delayed treatment of 1–2 days after the first sampling. Animal handling was in accordance with European (European Directive, 2010/63/EU) and German (German Animal Welfare Act [Tierschutzgesetz]) laws. The study design was presented to the responsible administrative federal state authorities for animal experiments and was not classified as an animal experiment (AZ 2684-05-015-22-206, Thuringian Federal State Office for Consumer Protection [Thüringer Landesamt für Verbraucherschutz]).

### 2.3 Faecal egg count via FLOTAC

The FLOTAC technique with 10 g of faeces and saturated sodium chloride (specific gravity 1.20) was performed individually for each sample as previously described (Cringoli et al., 2010). Both counting chambers of the FLOTAC device were examined, resulting in a multiplication factor of 1.

### 2.4 Egg isolation

The individual samples positive for *A. suum* or strongyle eggs were pooled per farm and age group. Sows and piglets, representing breeding farms, were pooled together. Detailed information about the egg isolation process was published previously (Fischer et al., 2024). In brief, eggs were separated from faeces particles using a kitchen sieve, a 25 μm sieve and centrifugation steps in saturated sodium chloride solution and tap water. In a final step the *A. suum* and strongyle eggs were separated based on their density with a sugar step gradient composed of four different sucrose solutions. Therefore 10 %, 25 %, 40 % and 60 % of a sucrose stock solution (60 g sugar in 40 mL distilled water) were diluted with water and layered in a 50 ml centrifugation tube. The egg suspension was overlaid and the gradients centrifuged at 1500×*g* for 5 min. The strongyle and ascarid eggs were visible between the 25 % and 40 % layers and the 40 % and 60 % layers, respectively. The eggs were then collected with a Pasteur pipette. The purified strongyle eggs were frozen in 300 μL aliquots (range 45-11,000 eggs) and ascarid eggs were kept at 4 °C until further processing.

### *2.5* In ovo *larval development assay*

**Fig. 1:**
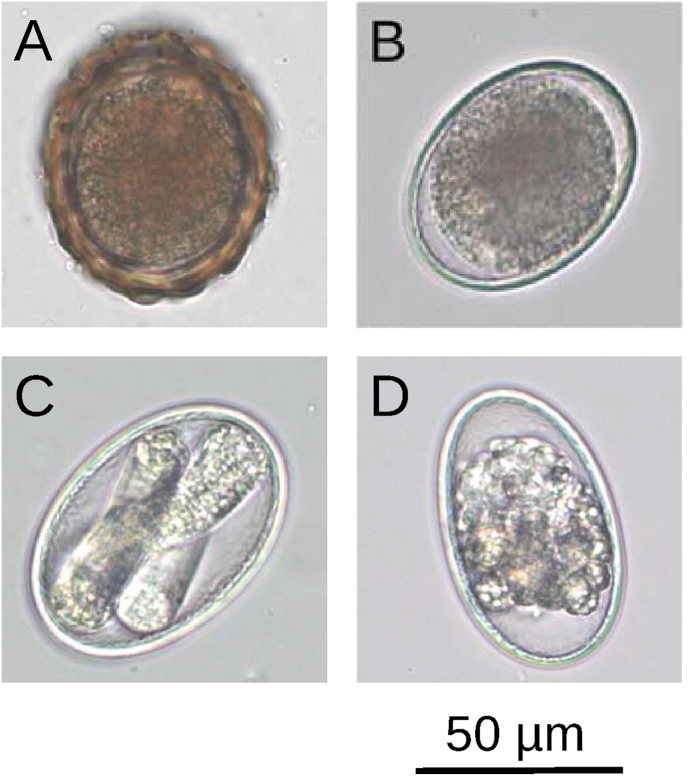
Microscopic pictures of *Ascaris suum* eggs at different stages of the *in ovo* Larval Development Assay. Ascarid eggs are shown after egg purification from faeces (A), after removal of the opaque outer layers with bleach (NaClO) (B), and after three weeks of incubation at 27 °C with completely developed L3 (C) and undeveloped egg (D).

The *in ovo* LDA is an *in vitro* assay, observing the larval development within the ascarid egg exposed to gradually increasing thiabendazole (TBZ) concentrations. The *in ovo* LDA for *A. suum* used in this study was established based on previously published LDAs examining *Parascaris* spp. and *A. galli* (Tydén et al., 2016; Tarbiat et al., 2017). In a first step, the eggs were washed three times with 0.9 % NaCl solution, to minimize contamination with bacteria or fungi from the faeces. In order to be able to examine the content of the ascarid eggs, the opaque outer layer of the undeveloped eggs was then removed with bleach (Fig. 1). For this purpose, 5 mL egg suspension was incubated with 15 mL of a sodium hypochlorite solution (8.75 mL NaOCl 12 % (v/v) plus 6.25 mL Aqua bidest preheated to 37 °C) at 37 °C for 3 min. The bleach was immediately removed from the eggs using a vacuum filtration unit (Filtropur V100, 0.2 μm, Sarstedt AG & Co. KG, Nümbrecht, Germany) and washed with 1.0–1.5 L of 0.9 % NaCl solution. The eggs were recovered from the top surface of the filtration unit and excess NaCl solution was removed via centrifugation (340×*g*, 5 min). Then, the eggs were resuspended in sodium phosphate buffer (10 mM, pH 7) at a concentration of 100–200 eggs/mL. Thiabendazole was dissolved in 100 % dimethyl sulfoxide (DMSO) resulting in a 200 mM stock solution. The stock solution was then diluted with DMSO in 1:2 and 1:1.5 dilution steps into 11 final TBZ concentrations. Of each dilution, 5 μL were pipetted into two wells of a 24-well test plate and 995 μL of the egg suspension was added to each well, resulting in concentrations of 62.50, 31.25, 15.63, 7.81, 5.21, 3.91, 2.60, 1.95, 0.98, 0.49 and 0.24 μM TBZ in 0.5 % DMSO in the wells. The vehicle control wells contained only DMSO in sodium phosphate buffer. The 24-well test plates were sealed with PARAFILM® to prevent evaporation and were incubated at 27 °C for 21 days in the incubator. Every 3–4 days each well was aerated using Pasteur pipettes to maximise larval development. After the incubation process, the total number of eggs and the number of eggs with a completely developed L3 were counted under the microscope.

### 2.6 Cultivation of Oesophagostomum spp. larvae (L3)

Faeces from a naturally infected sow were collected and a faecal culture (10 days, 25 °C and 80 % humidity) using sawdust was performed. The larvae were recovered and purified using the Baermann funnel system. Larvae were morphologically identified as *Oesophagostomum* spp. L3 (Deplazes et al., 2021) and 300 μL aliquots of larvae were frozen at −20 °C until further processing. ITS-2 PCR with primers NC1/NC2 (Gasser et al., 1993) followed by Sanger-Sequencing of transformed plasmids revealed the species *Oesophagostomum dentatum*. However, a mixed species infection cannot be completely excluded and the larvae will, therefore, be referred to as *Oesophagostomum* spp. larvae in the following text.

### 2.7 Genomic DNA extraction

Genomic DNA was extracted from individual adult specimens of *O. quadrispinulatum* stored in ethanol (originally obtained from the Department of Veterinary and Animal Sciences, University of Copenhagen, Denmark; Ethical approval by the Danish Animal Experimentation Inspectorate, license number 2005/561–1060), *Oesophagostomum* spp. larvae and strongyle eggs isolated from the participating farms. The ethanol was removed from the adult worms, the worms were washed with DEPC-treated water (PCR grade) and transferred individually into reaction tubes. The DNA of adult worms and larvae was extracted using the NucleoSpin® Tissue XS kit (Macherey-Nagel®, Düren, Germany) following the manufacturer's protocol. The samples were lysed using 8 μL proteinase K and 80 μL T1 buffer for 4 h at 56 °C in a shaker. Elution of the DNA was performed with 20 μL DEPC-treated water (PCR grade). From isolated strongyle eggs, DNA was extracted with the NucleoSpin® Soil kit (Macherey-Nagel®, Düren, Germany) according to the manufacturer's protocol. Aliquots (300 μL) from the egg isolation were lysed using 700 μL SL1 lysis buffer and mechanical beat beating in the kit's lysis tubes using a SpeedMill (Jena Bioscience). Elution of the DNA was performed with 50 μL DEPC-treated water (PCR grade). The obtained genomic DNA was frozen at −20 °C until further processing.

### 2.8 Nemabiome – sequencing of the internal transcribed spacer 2

Deep amplicon sequencing based on the ITS-2 gene region was carried out as described previously (Avramenko et al., 2015) modified according to Fischer et al. (2024). In brief, post-treatment genomic DNA was first amplified using 0.3 μM of each NC1/NC2 primer mix with Illumina adaptors in 50 μL 1 × Kapa HiFi HotStart Ready Mix using the following cycling conditions: initial denaturation at 95 °C for 2 min, followed by 30 cycles at 98 °C for 20 s, 62 °C for 15 s and 72 °C for 15 s, finally an extension period at 72 °C for 10 min. Library preparation and sequencing was performed as described for amplicons A and B.

Analysis of the ITS-2 deep amplicon sequencing data was carried out as described previously (Fischer et al., 2024). IdTaxa was trained with the nemabiome ITS-2 database v1.5.0 (Workentine et al., 2020).

### 2.9 Isotype-1 β-tubulin gene region

#### 2.9.1 Isotype-1 reference β-tubulin gene sequences

First, partial isotype-1 β-tubulin gene sequences were generated from DNA isolated from *Oesophagostomum* spp. larvae. Primer sequences are provided in Table S1. All primers were obtained from Integrated DNA Technologies (IDT, Inc., Leuven, Belgium). The longest continuous sequences were amplified with the forward primers Beta_F3, Beta_F1 and one reverse primer Beta_R (Table S1) from codons 85 and 117 to codon 218, respectively. Primer sequences were generated based on sequences provided in the BioProject PRJNA72579 (Wormbase ParaSite). PCR reactions contained 2 μL template DNA, 0.5 μM of each primer, 0.4 U Phusion Hot Start II High-fidelity DNA polymerase (Thermo Scientific) and 1 M betaine in 20 μL 1 × HF buffer. The 2-step PCR protocol included initial denaturation at 98 °C, 30 cycles of denaturation at 98 °C for 10 s, and annealing and extension at 68 °C for 1.5 min, followed by a final extension at 72 °C for 10 min. The PCR products were analysed by agarose gel electrophoresis and cloned using the StrataClone Blunt PCR Cloning Kit (Agilent, Waldbronn, Germany) and submitted to Sanger sequencing to LGC Genomics (Berlin) (GenBank accession numbers PV442195-PV442203).

#### 2.9.2 Phylogenetic analysis of the isotype-1 β-tubulin gene

The coding sequences of the β-tubulin gene of *Oesophagostomum* spp. (generated from primer pair Beta_F3/Beta_R and Beta_F1/Beta_R amplicons), of *Parascaris univalens*, *A. suum*, *Caenorhabditis elegans* and *Haemonchus contortus* previously analysed by Özben et al. (2022), and of *Ancylostoma duodenale* (GenBank accession numbers DQ055415.1, EF392850.1), *Ancylostoma caninum* (DQ059758.1, EU876852.1), *Necator americanus* (EU182348.1, EF392851.1) and *Cylicocyclus nassatus* (AF283765.1) were aligned codon-wise using MUSCLE in Mega v7.0.26 (Kumar et al., 2016). The degree of saturation was estimated with the substitution saturation test (Xia et al., 2003) for codon positions 1 and 2, and codon position 3 separately with DAMBE v7.0.35 (Xia, 2018). Since a high substitution saturation was observed for codon position 3, codon-based substitution models were chosen instead of fitting separate DNA substitution models for the different codon positions. The IQ-TREE web server v1.6.12 (Nguyen et al., 2015; Trifinopoulos et al., 2016) was used to calculate the phylogenetic tree. ModelFinder (Kalyaanamoorthy et al., 2017) with inclusion of „FreeRate heterogeneity“ models was used for substitution model selection and the branch support was calculated using ultrafast bootstrapping (Hoang et al., 2018) with 1000 bootstrapped alignments and the Shimodaira-Hasegawa-like approximate likelihood ratio test (SH-aLRT) (Guindon et al., 2010). FigTree v1.4.4 (Rambaut, 2007) was used in order to generate the visualisation of the final phylogenetic tree.

#### 2.9.3 Deep amplicon sequencing of isotype-1 β-tubulin amplicons A and B

In an initial PCR, two regions of the isotype-1 β-tubulin gene region of *Oesophagostomum* spp. were targeted for deep amplicon sequencing. Amplicon A, targeting codons 134 and 167, was amplified with the primers Beta_Exon3-4_Ospp_F 5ˈ-CCCGATAACTACGTGTTTGGA-3ˈ and Beta_Exon3-4_Ospp_R 5ˈ-CTTGGGTGACGGTACIAC-3ˈ. Amplicon B, targeting codons 198 and 200, was amplified with the primers Beta_Exon5-6_Ospp_F 5ˈ- GTCTCGGACACTGTGGTAGAGCCATACAAT-3ˈ and Beta_Exon5-6_Ospp_R 5ˈ- AATTGACCAGGGAAGCGAAGGCAAGTAGTT-3ˈ. Each primer was modified by adding sequences of Illumina adapters at the 5ˈ-end and with 0–3 additional random bases (N) (Table S1) as described previously (Avramenko et al., 2015, 2019; Redman et al., 2019) and all four versions of one primer were mixed in equal molar amounts. Two separate amplifications for amplicons A and B were carried out with each PCR reaction containing 8 μL template DNA and 0.3 μM of the forward and reverse primer mix in 50 μL 1 × Kapa HiFi HotStart ReadyMix (Roche, Mannheim, Germany). The PCR protocol included initial denaturation at 95 °C for 2 min, 35 cycles of denaturation at 98 °C for 20 s, annealing at 54.5 °C (amplicon A) or 59 °C (amplicon B) for 15 s and elongation at 72 °C for 15 s, followed by a final extension at 72 °C for 2 min.

For library preparation, the PCR products were cleaned with AMPure XP Magnetic Beads (Beckman Coulter GmbH, Krefeld, Germany) and eluted in 40 μL 10 mM Tris-HCl (pH 8.0), quantified with the Qubit dsDNA HS Assay Kit (Thermo Fisher Scientific, Darmstadt, Germany) on a Qubit 4 Fluorometer (Thermo Fisher Scientific), following the manufacturer's protocol. 10–12 ng purified PCR products were then indexed (P5/P7) by limited cycle PCR amplification, with 2.5 μM dual index primers (Illumina, San Diego, California, USA) in 25 μL of Kapa HiFi ready mix. PCR conditions were chosen as followed: 98 °C for 45 s, 7 cycles of 98 °C for 20 s, 63 °C for 20 s and 72 °C for 20 s, followed by a final extension at 72 °C for 2 min. The indexed PCR products where then again cleaned with magnetic beads with an elution volume of 25 μL 10 mM Tris-HCl (pH 8.0), the concentration was quantified on the Qubit 4 Fluorometer, diluted to 4 nM in 10 mM Tris-HCl buffer, pooled according to the Illumina protocol and finally run on a MiSeq benchtop sequencer (V3, 2x300 bp, Illumina).

To test the ability of the method to detect SNPs, gBlocks® Gene Fragments (IDT, USA) of amplicons A and B with and without SNPs at the codons 134, 167, 198 and 200 of *O. dentatum* and *O. quadrispinulatum* were mixed in three different ratios (susceptible/resistant: 20/80 %, 50/50 % and 80/20 %) and used as template for the PCRs and deep amplicon sequencing (8 μL, 10^4^ copies/μL; PCR conditions as described).

#### *2.9.4* *Bioinformatic analysis* – *Dada2*

Sequence reads from the amplicons A and B isotype-1 β-tubulin deep amplicon sequence analysis were demultiplexed by the MiSeq software and adapters as well as primers were removed using cutadapt v3.5-1 (Martin, 2011). The analysis pipeline is described in detail on the nemabiome web page (https://www.nemabiome.ca/; last visited: 14.06.23) using the R package dada2 v1.28.0 (Callahan et al., 2016), which was used to filter reads with maximum expected errors of two and five for forward and reverse sequences, respectively. Truncation of reads to a quality score of a maximum of two expected errors per read was performed. Sequences were then denoised, based on an error profile generated by the dada2 algorithm and merged to full amplicon sequences. Finally, chimeras were removed using dada2. A taxonomic classification was sought for the resulting amplicon sequence variants (ASVs) using IdTaxa from the R package DECIPHER v2.28.0 (Wright, 2016; Murali et al., 2018) with the threshold set at 60 and 100 bootstrap repeats. Amplicon A and B sequences generated via Sanger-sequencing based on *Oesophagostomum* spp. larval DNA and adult *O. quadrispinulatum* DNA (reference sequences) were used to train IdTaxa. Additionally, a BLASTx (Altschul, 2014) of the ASVs against a *Haemonchus contortus isotype-1* β-tubulin protein sequence (GenBank accession number ABM92348.1) was carried out. Further 17 ASVs of amplicon B identified as β-tubulin sequences, where then aligned with the reference sequences and showed at least 90 % identity with the *Oesophagostomum* amplicon B. High variability in the intron sequence in amplicon B caused this low level of identity. Subsequently, these identified sequences were incorporated into the reference sequence database. The amplicon A and B sequences used for idTaxa training are provided in the Data S2 and Data S3, respectively. Amplicon sequence variants were then checked manually for SNPs on codon positions 134, 167, 198 and 200. Raw deep sequencing data of the β-tubulin gene regions are available in the short read archive (SRA) of GenBank under the BioProject ID PRJNA1201733.

#### 2.9.5 Haploid network

To create a haploid network of isotype-1 β-tubulin sequences, the ASVs obtained from sequencing of amplicons A and B were aligned separately using MAFFT (Katoh et al., 2019) with the Q-INS-i and the “leave gappy regions” option. A traits file was generated with the number of reads per sample and ASV. In order to be able to show ASVs with high read counts and to not loose ASVs with read counts <100 at the same time, the number of reads was divided by 100 and one was added (+1). Finally, the software PopART (Leigh and Bryant, 2015) was used to create a TCS haplotype network (Templeton et al., 1992; Clement et al., 2000). Due to the intron region present in both amplicons and the resulting gaps in the alignment, few of the ASVs (Amplicon A: ASV15; Amplicon B: ASV6, ASV18, ASV26, ASV49, ASV67 and ASV163) were excluded from the haplotype network by the PopART algorithm. For visualisation purposes, the excluded ASVs were processed separately with PopART and manually included in the graph next to the ASVs from which they only differed by insertions/deletions.

### 2.10 Statistical analyses

#### 2.10.1 Faecal egg count reduction with credible intervals

The package eggCounts v2.3-2 (Torgerson et al., 2014; Wang et al., 2018) was used in R v4.1.3 accessed through the graphical user interface RStudio 2023.06.1 to calculate the estimate for the FECR and its 90 % credible intervals (CrIs). Calculations were performed for animal groups with at least 200 eggs counted under the microscope for pre-treatment samples in order to comply with the W.A.A.V.P. guideline research protocol for BZ and *O. dentatum* (Kaplan et al., 2023). Due to coprophagy, low *A. suum* egg counts are often suspected to be false positive. Consequently, two analyses of the FECRT for *A. suum* were conducted, one including all egg counts and one considering EPG values < 200 pre- and post-treatment as negative (i.e., they were set to zero). At least nine animals were included per group and groups with only one positive animal pre-treatment were excluded from further calculations. For all but Farm 14, where a reliable identification of the animals was not possible after treatment, the egg counts algorithm was used with paired data. For all calculations a group wide identical efficacy was assumed (no individual efficacy on individual animal level) and “zero inflation = FALSE” was applied. The 90 % CrIs were calculated as described previously (Krücken et al., 2024) using the stan2mcmc function of eggCounts to convert the stan object created by eggCounts into an mcmc object and the HPDinterval function from the coda v0.19-4 package to extract the highest posterior density (HPD) intervals from the posterior distributions. Additionally, Wilcoxon matched-pairs signed rank tests and a Mann-Whitney *U* Test were performed using the function wilcox.test() from the R base package stats v4.4.1 to estimate the effect of the TBZ treatment across all farms and on Farm 14, respectively.

#### 2.10.2 EC_50_ value calculations for the in ovo larval development assay

GraphPad Prism 5.03 software for Windows was used to calculate concentration-response curves and statistical analysis from the LDA data (including the EC_50_, the 95 % confidence intervals (CI) and R^2^ values). A non-linear regression (four-parameter logistic equation with a variable slope) was performed after transforming the drug concentrations into its logarithm (X corresponds to log_10_X).

#### 2.10.3 Effects of fenbendazole treatment on species composition

The effects of BZ treatment on species composition were calculated based on the percentages of ITS-2 deep amplicon sequencing reads assigned to *O. dentatum* and *O. quadrispinulatum*, for Farms 10 and 2. A Fisher's exact test with mid-p correction was calculated using the function tab2by2.test() from the R package epitools v0.5–10.1 (Aragon, 2020). Bonferroni correction was performed using p.adjust() from the R base package stats v4.4.1.

#### 2.10.4 Linear regression analysis

GraphPad Prism 5.03 software for Windows was used to calculate linear regression models based on the deep amplicon sequencing results of the artificial gBlocks® Gene Fragments.

## 3 Results

### 3.1 Farms

A total of 17 farms were visited between February 2022 and March 2023. In the following, the farms and animal groups will be indicated as followed: ‘F’ for farm, individual farm number corresponding to the numbering in Fischer et al. (2024), and a capital letter indicating the age classes as followed: ‘P’ for piglets, ‘F’ for fatteners, ‘S’ for sows and ‘B’ for breeding (piglets and sows combined). Farms where no tests (FECRT, LDA, NGS) could be performed were not further mentioned in this publication. All but three farms were EU certified organic farms. The non-organic farms included farms F2F and F3P (NEULAND farms, (Verein für tiergerechte und umweltschonende Nutztierhaltung e.V, 2024)) and F14P, which was in the process to converting to an EU certified organic farm. For more details such as geographical distribution of the farms and farming system see Fischer et al. (2024).

### 3.2 Regular treatment practices on the farms

Routine anthelmintic treatment was carried out using BZs and MLs on twelve and three farms, respectively. Benzimidazoles were almost exclusively administrated via feed (Farms F1, F2, F4, F5, F6, F10, F12, F13a, F15 and F16) and only one farm administrated them via the drinking water (F10). In contrast, only one farm (F14P) treated its animals with ML via feed and two farms (F3S and F13bS) via injection. No regular anthelmintic treatment was performed on F11P and F13aF. The remaining farms treated at least one age group regularly. Piglets were the least commonly treated group, with only 50 % treatment rate (5 out of 10 farms). They were generally treated once before moving to the fattening units. Treatment of fatteners was administered on six out of ten farms, with variations in the treatment intervals. Two farms treated every six weeks, one farm 1–3 times and three farms once in the fattening period. Sows were treated most commonly (7 out of 10 farms), with varying frequencies from every two years to three times a year. Most common was a treatment interval of two times per year (three farms) and two farms treated once prepartum.

The duration of individual treatments fluctuated between one and six days, with one and three days being most common. However, farmers reported that they had utilised the intended quantity for a single day for an extended treatment period of three or even six days.

Two farms stated to change the anthelmintic on a regular basis. However, one of the farms switched between active components of the same class (FBZ and flubendazole) in the same fattening period and the other one was not able to give any specific information about the products used, due to a recent change of the farm management.

### 3.3 Egg count results

Details on number of tested animals per farm, age class, median EPG pre- and post-treatment, number of negative tested animals (EPG = 0) and number of animals with an egg count <200 EPG can be found in Table 1 for strongyles and Table 2 for *A. suum*. The raw pre- and post-treatment EPGs are provided in Table S4.

**Table 1:** Pre- and post-treatment egg count data and FECRT results for strongyles.

Farm^a^	n	Scale	n_eggs_	Pre-treatment		Post-treatment		FECR with 90 CrIs [%]
				n_0_	EPG	n_0_	EPG	
**F2F**	22	Yes	10,000^b^	0	131 (15–806)	21	0 (0–1)	99.9 (99.9–100)
**F3P**	10	No	7000	4	2.5 (0–239)	all	0	99.9 (99.9–100)
**F5F**	17	Yes	1600	2	33 (0–178)	all	0	99.9 (99.7–100)
**F7F**	22	Yes	5200	10	3 (0–260)	all	0	99.9 (99.5–100)
**F10S**	10	Yes	–	0	390.5 (0–3172)	8	0 (0–13)	99.8 (99.7–100)
**F10P**	25	Yes	–	12	3 (0–255)	all	0	99.9 (99.8–100)
**F10B**	35	Yes	4300; 130^c^	12	35 (0–3172)	33	0 (0–13)	99.8 (99.7–99.9)
**F11P**	9	No	–	0	3100 (204–5871)	all	0	100 (99.9–100)
**F11B**	13	No	4500	0	3561.5 (204–5871)	all	0	100 (99.9–100)
**F14P**	21/17^d^	No	1900	0	132 (6–1098)	16	0 (0–5)	99.8 (99.6–99.9)^d^

Farm, farm number with the capital letter in the end indicating the age class as followed: P for piglets, F for fatteners, S for sows and B for breeding (piglets and sows combined); n, number of individual paired pre- and post-treatment egg counts; Yes, scale was used for weight; No, weight was estimated; n_eggs_, number of purified eggs used for Next Generation Sequencing (deep amplicon sequencing of β-tubulin isotype 1 and ITS-2 gene regions); n_0_, number of animals with an egg count of 0 EPG; EPG, median egg counts in eggs per gram faeces with range, with “all” indicating that all egg counts were negative/ = 0; FECR with 90 % CrIs, Faecal Egg Count Reduction Test estimates with the 90 % credible intervals.

a Farms, where no FECRT could be calculated were not included in this table (F4S, F6S, F6P, F9P, F9F, F13aF, F15F).

b Strongyle eggs from Farm 2 were purified on two occasions pre-treatment (9000 & 11,000 eggs) and once post-treatment (unknown, very low number of parasite eggs, no eggs could be counted after purification).

c Strongyle eggs from Farm 10 were purified pre-treatment (4,300) and post-treatment (130).

d FECRT calculations based on unpaired egg count data, with 21 pre-treatment and 17 post-treatment individual egg counts.

**Table 2:** Pre- and post-treatment egg count data, FECRT and *in ovo* LDA results for *Ascaris suum*.

Farm^a^	n	Scale	Pre-treatment		Post-treatment		FECR_all_ with 90 CrIs [%]	FECR_>200_ with 90 CrIs [%]	*in ovo* LDA		
			n _<_ _200_ (n_0_)	EPG	n _<_ _200_ (n_0_)	EPG			Eggs/well (range)	TBZ EC_50_ (95 % CI) [μM]	R^2^
F2F	22/30^b^	Yes	30 (19)	0 (0–164)	30 (19)	0 (0–12)	84.3 (79.8–88.3)	–	228 (188–258)	1.504 (1.446–1.566)	0.9983
F3P	10	No	5(1)	108.5 (0–1101)	10 (7)	0 (0–2)	99.9 (99.7–100)	99.9 (99.9–100)^c^	193 (174–246)	3.356 (3.272–3.442)	0.998
F4S	13	–	13(9)	0 (0–71)	–	–	–	–	146 (110–165)	2.692 (2.630–2.757)	0.9965
F5F	17	Yes	16 (12)	0 (0–205)	all	0	99.9 (98.9–100)	99.8 (98.8–100)^c^	216 (154–240)	1.861 (1.792–1.934)	0.9968
F6S	8	–	8 (6)	0 (0–128)	–	–	–	–	163 (133–207)	2.622 (2.593–2.652)	0.9987
F7F	22	Yes	14 (4)	16.5 (0–6496)	all	0	100 (99.9–100)	100 (99.9–100)	227 (155–269)^e^	2.102 (2.062–2.144)	0.9983
F8F	19	Yes	16 (12)	0 (0–5596)	all	0	100 (99.9–100)	100 (99.9–100)	209 (170–296)^e^	1.816 (1.800–1.832)	0.9998
F9P	14	No	9 (6)	47.5 (0–582)	14 (11)	0 (0–5)	99.6 (99.4–99.8)	99.9 (99.9–100)	167 (142–248)	2.287 (2.231–2.344)	0.9978
F9F	27	No	14 (2)	213 (0–1720)	27 (15)	0 (0–6)	99.8 (99.6–99.8)	100 (99.9–100)	237 (185–292)	2.474 (2.453–2.495)	0.9995
F11P	9	No	7(3)	23 (0–1076)	8(3)	0 (0–460)	72.9 (70.5–75.2)	68.6 (65.5–71.2)	229 (172–265)	1.52 (1.491–1.549)	0.9997
F11B	13	No	10 (6)	2 (0–1076)	12 (12)	0 (0–460)	73.1 (70.6–75.4)	99.9 (99.8–100)			
F13aF	19	Yes	18 (8)	2 (0–852)	19 (10)	0 (0–4)	97.6 (96.7–98.4)	99.9 (99.7–100)	70 (51–89)	2.947 (2.892–3.004)	0.9989
F14P	21/17^d^	No	16(14)	0 (0–1342)	17 (15)	0 (0–3)	96.2 (24.1–100)^d^	94.7 (16.7–100)^d^			
F15F	27	No	26 (17)	0 (0–224)	27 (20)	0 (0–4)	93.7 (90.8–96.4)	99.9 (98.9–100)^c^	148 (107–174)	2.024 (2.010–2.039)	0.9995

Farm, farm number with the capital letter in the end indicating the age class as followed: P for piglets, F for fatteners, S for sows and B for breeding (piglets and sows combined); P, piglets; F, fatteners; S, sows; n, number of individual paired pre- and post-treatment egg counts; Yes, scale was used for weight; No, weight was estimated; neggs, number of purified eggs used for Next Generation Sequencing (deep amplicon sequencing of β-tubulin isotype 1 and ITS-2 gene regions); n0, number of animals with an egg count of 0 EPG; n < 200, number of animals with an egg count <200 EPG, including 0 EPG; EPG, median egg counts in eggs per gram faeces with range, with “all” indicating that all egg counts were negative/ = 0; FECR_all_, Faecal Egg Count Reduction Test calculated including all egg counts (incl. <200 EPG); FECR_>200_, FECRT calculations without egg counts <200 EPG; in ovo LDA, in ovo Larval Development Assay; TBZ, thiabendazole; CI, confidence interval; EC_50_, half maximal effective concentration. EC_50_ values, 95 % CI and R^2^ were computed with GraphPad Prism software.

a Farms, where no FECRT or *in ovo* LDA could be calculated were not included in this table (F6P, F10S, F10P, F10B, F11S).

b The FECRT was performed twice on Farm 2. For strongyles 22 and for A. suum 30 individual paired egg counts were included in the FECRT calculations.

c Calculations based on one positive animal (EPG >200) before treatment.

d FECRT calculations based on unpaired egg count data, with 21 pre-treatment and 17 post-treatment individual egg counts.

e Only one instead of two replicates for the negative control, due to the lack of eggs.

### 3.4 Faecal egg count reduction test results

**Fig. 2:**
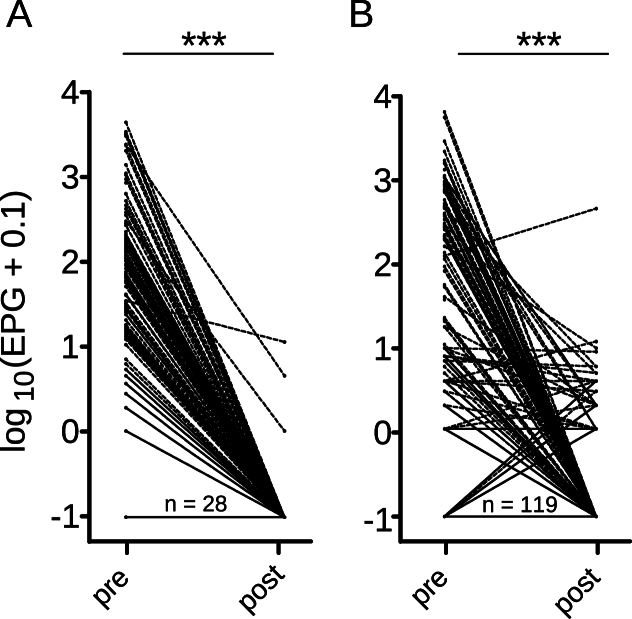
Paired eggs per gram faeces (EPG) pre and post fenbendazole treatment of strongyles (A) and ascarids (B) across all farms, respectively. For visualisation purposes 0.1 was added to the EPG and then numbers were logarithmised. ∗∗∗, p < 0.001. Effects of treatments were analysed using a Wilcoxon matched-pairs signed rank test. The unpaired EPG data from Farm 14 were included without lines between pre- and post-treatment pairs, since it was not possible to unequivocally identify them. Number (n) of animals with an EPG = 0 are indicated in the graphic.

Of 604 animals sampled, 276 individuals from different age groups were included in the FECRT calculations. The results of the FECRT calculations for strongyles and *A. suum* are shown in Table 1, Table 2, respectively. For strongyles as well as for *A. suum* the FECRT was calculated for each age group separately. For two farms (Farm 10 and 11), the FECRT was additionally calculated for sows and piglets combined in order to increase the number of animals included in the calculations (Table 1, Table 2). When considering all egg counts, including egg counts <200 EPG for *A. suum*, FECRT estimates for the age group piglets and sows, as well as for piglets and sows combined (breeding) showed very similar results (Table 2). Between 9 and 36 individuals per animal group were included for FECRT calculations. Except for three farms for FECRT calculations without EPGs <200 (F3P, F5F, F13aF), animal groups with only one positive animal pre-treatment were not considered for FECRT calculations. The paired pre- and post-treatment strongyle and *A. suum* EPGs of all farms are visualised in Fig. 2A and B, respectively. Overall, a significant reduction (Wilcoxon matched-pairs signed rank test, p < 0.001, Fig. 2A and B) of the strongyle and *A. suum* egg counts across all farms was observed. Differences in the pre- and post-treatment EPGs of strongyles and *A. suum* across all farms can be noticed in Fig. 2A and B.

#### 3.4.1 Faecal egg count reduction tests for strongyles

In total, seven strongyle FECRTs with a total of 117 paired and 21 unpaired (F14P) individual egg counts were analysed. The EPG key figures are, pre- and post-treatment, respectively: median 55 and 0 EPG; mean 404.86 and 0.18 EPG; range 0–5871 and 0–13 EPG, percentage of animals with negative egg counts 20.3 % and 94.2 %.

The W.A.A.V.P. FECRT research protocol for *O. dentatum* (Kaplan et al., 2023) was adhered to for all FECRT. Pigs included in the FECR calculations had not been treated for at least 9 weeks. All FECR values and the lower 90 % CrIs were higher than the target efficacy for *O. dentatum* (99 %) and ranged from 99.8 % to 100 % (Table 1). The FECRT results show that FBZ was still fully active on all seven farms.

#### 3.4.2 Faecal egg count reduction test for Ascaris suum

In total, 219 paired and 21 unpaired (F14P) individual *A. suum* egg counts were analysed (Table 2). The following pre- and post-treatment EPG key figures were observed, respectively: median 1 and 0 EPG, mean 194.41 and 2.51 EPG, range 0–6496 and 0–460 EPG, percentage of animals with negative egg counts 49.6 % and 77.5 %. It is remarkable, that for *A. suum* the total number of negative egg counts only increased from 49.6 % pre-to 78.8 % post-treatment.

The W.A.A.V.P. guideline for using the FECRT does not provide any research protocols or target efficacies for *A. suum* FECR calculations (Kaplan et al., 2023). Therefore, for the present study the target efficacy was set conservatively at 95 %, to reduce the number of worm populations falsely interpreted as resistant. Evaluation of FBZ efficacy revealed the following FECR estimates below 95 %, calculated with and without egg counts <200 set to zero, respectively: F2F with an estimate of 84.3 % (90 % CrIs 79.8–88.3 %) (all egg counts were <200, therefore only one estimate was available), F11P 72.9 % (90 % CrIs 70.5–75.2 %) and 68.6 % (90 % CrIs 65.6–71.2 %), F11B 73.1 % (90 % CrIs 70.6–75.4 %) and 99.9 % (90 % CrIs 99.8–100 %), F14P with 96.2 % (90 % CrIs 24.1–100 %) and 94.7 % (90 % CrIs 16.7–100 %), and F15F with 93.7 % (90 % CrIs 90.8–96.4 %) and 99.9 % (90 % CrIs 98.9–100 %). The results show, that in most cases the FECR calculations without egg counts <200 EPG increased the FECR estimates and the 90 % CrIs. However, in case of F5F, F11P and F14P (unpaired EPG data), the FECR estimates and the 90 % CrIs decreased (Table 2). Except for F2F, where the pigs were treated seven weeks prior to the first sampling, the recommendations from Kaplan et al. (2023) (at least 8 weeks) were met.

### 3.5 In ovo larval development assay results

**Fig. 3:**
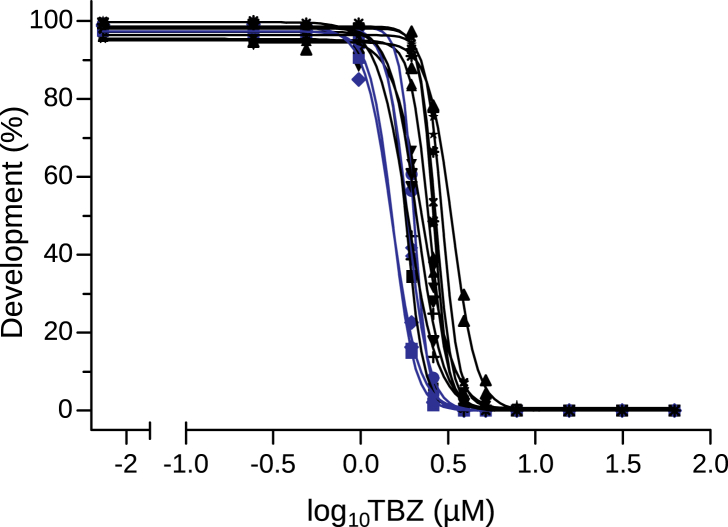
*Ascaris suum in ovo* Larval Development Assay results. Concentration-response curves were computed with the GraphPad Prism software, based on developed L3 per well (%) and log_10_-transformed thiabendazole (TBZ) concentrations (from 62.5 to 0.24 μM TBZ in 0.5 % dimethyl sulfoxide) in two replicates. Farms with faecal egg count reduction estimates <95 % are highlighted in blue.

The LDA was performed for 13 *A. suum* egg samples from 12 different farms (Table 2). For all tests, a median of 194 eggs/well (range 51–269 eggs/well) was calculated. Individual egg counts per well, EC_50_ values and R^2^ are shown in Table 2. The computed EC_50_ values range from 1.50 to 3.36 μM TBZ with a median and mean of 2.10 μM and 2.24 μM TBZ, respectively. The concentration-response curves are visualised in Fig. 3. Farms that showed FECRT estimates and 90 % CrIs indicating AR, namely F2F, F11P and F15F were shown to have EC_50_ values (1.504, 1.52 and 2.024 μM TBZ, respectively) below the mean EC_50_ value of 2.236 μM TBZ (Table 2).

### 3.6 Fenbendazole treatment effects on strongyle species composition

**Fig. 4:**
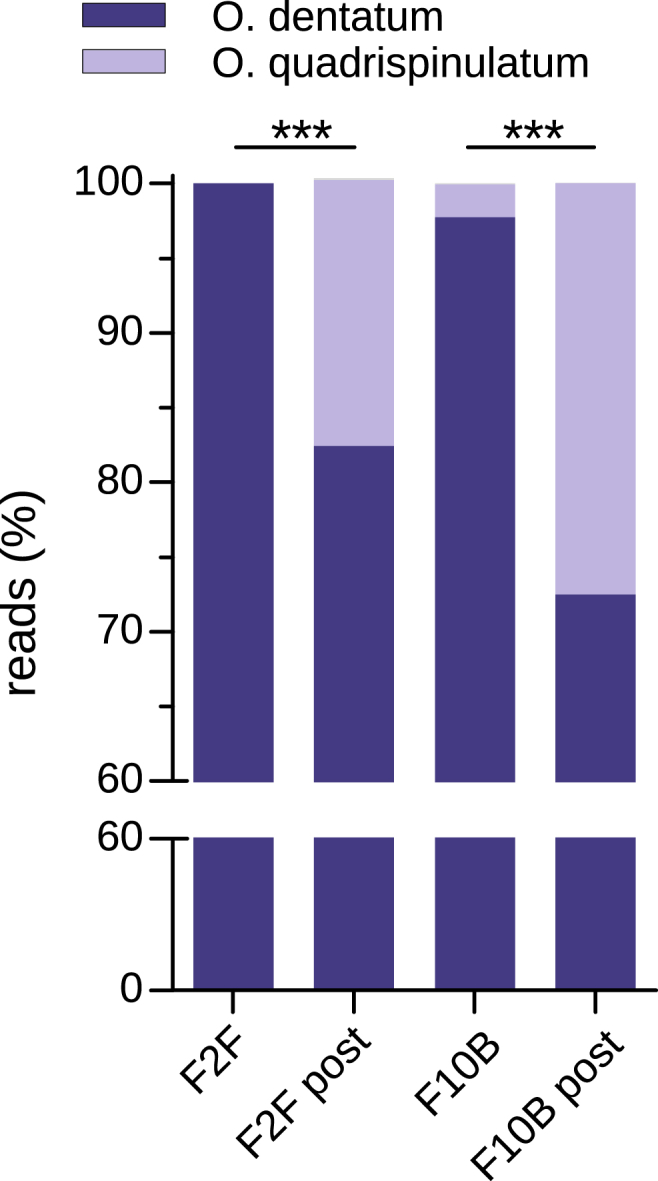
Proportions (%) of the ITS-2 deep amplicon sequencing reads assigned to the species *Oesophagostomum dentatum* and *O. quadrispinulatum* pre- and post-treatment from Farms F2F and F10B, respectively. The proportion of *O. quadrispinulatum* increased significantly after treatment (Fisher's exact test with mid-p correction); ∗∗∗, p < 0.001.

A total of 71,301 and 36,348 paired end reads were generated for the post-treatment samples of F2F and F10B, respectively. The final number of reads after processing with the dada2 pipeline and species assignment with IdTaxa were 51,443 reads for sample 2.1 and 26,460 reads for sample 10. The pre-treatment results were previously published (Fischer et al., 2024). The pre- and post-treatment distributions of the ASVs and the species assignment are shown in Fig. 4. Overall, the proportion of *O. quadrispinulatum* increased significantly (Fisher's exact test with mid-p correction, p < 0.001) after treatment. In the post-treatment samples three ASVs that were not present pre-treatment, assigned to *O. dentatum* (ASVs 27 and 37) and *O. quadrispinulatum* (ASV69), were detected (GenBank PP785341-PP785343).

### 3.7 Detection of benzimidazole-resistance polymorphisms in isotype-1 β-tubulin

#### 3.7.1 Identification of the amplified β-tubulin isotype

The amplicon A and B sequences are not sufficiently long or distinct to allow for a reliable identification of the isotype. Instead, the isotype of the longer reference sequences (generated with primers Bet_F3, Beta_F1 and Beta_R; Table S1) used for primer design was determined using phylogenetic analysis. Sequences of primer pair Beta_F3/Beta_R and Beta_F1/Beta_R cover the codons 94–207 and 128–207 of isotype-1 β-tubulin gene, respectively (reference sequence: ABM92348.1). In order to ensure that the amplified tubulin fragments correspond to the isotype-1 β-tubulin gene and not to one of its paralogs, the generated reference sequences *Oesophagostomum* spp. were analysed together with sequences of various β-tubulin isotypes from different nematode species. In the codon-based substitution model maximum-likelihood phylogenetic tree (Fig. S5), the generated *Oesophagostomum* sequences are situated in a well to highly supported monophyletic cluster of isotype-1 sequences. The two isotype-2 sequences formed their own cluster, separated from the isotype-1 cluster. Each, the *C. elegans* isotype-1 and 2 and the ben-1 isotype are also in a separate cluster. Most of the β-tubulins of clade III nematodes (*A. suum*, *A. lumbricoides*, *P. univalens*), isotypes A, B, C, D and G, form their own large cluster. Moreover, there are separate clusters for the mec-7 like and tbb-4 like isotypes.

#### 3.7.2 Frequencies of benzimidazole resistance-associated polymorphisms in Oesophagostomum spp. from pigs

Between 119 and 16,200 (median 10,529) paired end reads were generated for amplicon A (targeting codons 134 and 167). The number of reads obtained per sample after trimming, filtering and merging was between 44 and 13,584 (median 8711). The filtering statistics of the raw reads for amplicons A and B are detailed in Table S6. For the sample of F13aF no ASVs could be assigned to isotype-1 β-tubulin of *Oesophagostomum* spp. and all reads were, therefore, excluded from further analysis. For the rest of the samples, between 0 and 65 % of the reads (median 4 %) were likewise excluded from further analysis.

Between 264 and 31,634 (median 19,807) paired end reads were generated for amplicon B (targeting codons 198 and 200). The final number of reads after trimming, filtering and merging ranged between 208 and 26,987 (median 18,524) (see Table S6 for details). Between 0 % (F13aF) and 72 % of the reads (median 0.6 %) could not be assigned to isotype-1 β-tubulin of *Oesophagostomum* spp. and were, therefore, excluded from further analysis.

To determine if the deep amplicon sequencing approach is able to reliably detect SNPs in the *Oesophagostomum* spp. amplicons, artificial gene fragments containing Q134H, F167Y, E198A and F200Y and fragments without SNPs were mixed in different ratios and used as template for deep amplicon sequencing. The results of a linear regression analysis with two values per expected SNP occurrences are visualised in Fig. S7. The computed slopes of the regression lines for Q134H, F167Y, E198A and F200Y ranged from 0.902 to 1.006 and did not significantly differ from 1 (p values: 0.891, 0.945, 0.130 and 0.130, respectively; GraphPad Prism 5.03). The corresponding computed R^2^ were 0.9787, 0.9776, 0.9754 and 0.9754 for Q134H, F167Y, E198A and F200Y, respectively. The Y intercept ranged from −7.79 to 4.44 (for more details see Fig. S7).

Overall, no SNPs associated with resistance were detected in any pre- or post-treatment sample. A total of 18 amplicon A ASVs could be assigned to *Oesophagostomum* spp*.* Due to high similarities in the *Oesophaogostomum* spp. and *O. quadrispinulatum* sequences, a distinction between species was not possible for Amplicon A. At codon position 134, a silent mutation (CAA → CAG, both versions coding for Q/glutamine) could be observed in two samples with a low frequency of 0.41 % and 0.66 % on F2F and F14P, respectively. At codon position 167, the TTC coding for phenylalanine and associated with BZ-susceptibility was detected exclusively in all samples.

For amplicon B, the difference between the *O. quadrispinulatum* and *Oesophagostomum* spp. (presumably *O. dentatum*) was large enough to allow differentiation for at least some of the ASVs. A total of 33 ASVs was assigned to the genus *Oesophagostomum*, of which 7 were assigned to *O. quadrispinulatum*. The median percentage of reads assigned to *Oesophagostomum* spp. and *O. quadrispinulatum* was 99.6 % (range 79–100 %) and 0.4 % (0–21 %), respectively. At codon position 198, two version coding for glutamate (susceptible) were detected, with GAG (median 89.4 %, range 1.8–100 %) being more frequent than GAA (median 10.6 %, range 0–98.2 %). At codon position 200, only the variant TTC coding for phenylalanine was detected in all samples.

**Fig. 5:**
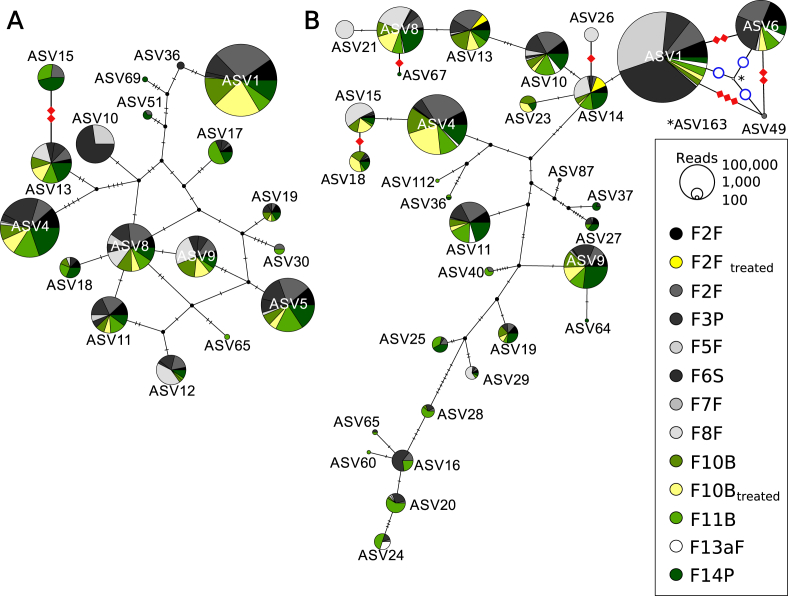
Haplotype network for amplicons A (A) and B (B) for isotype-1 β-tubulin amplicon sequence variants (ASVs). The size of the circles indicates the number of reads assigned to the ASV and the colours the farm. Colours additionally indicate post-treatment samples (yellow; F2F and F10B), farms with access to pasture (green; F10B, F11B, F14P) and farms with concrete outdoor pens (grey, black, white; F2F, F3P, F5F, F6S, F7F, F8F, F13aF). Strongyle eggs were isolated twice pre-treatment on F2F. For visualisation purposes, the number of reads was divided by 100 and 1 was added. Red squares indicate manually included ASVs and the number of nucleotide differences between the connected ASVs. The intron region in amplicon B is missing entirely in ASV163 and is shown as a blue circle in the graph. The remaining sequence of ASV163 (155 bp) is identical with ASV1, ASV6 and ASV49. Only 7 reads of ASV163 were detected on Farm 5.

The distribution and abundance of amplicons A and B ASVs across farms are shown in two haploid networks in Fig. 5.

## 4 Discussion

In the current study no BZ-resistance in strongyles or ascarids was detected on German alternative pig farms using the combination of FECRT and deep amplicon sequencing or FECRT and *in ovo* LDA, respectively. Overall, there are only few reports on AR in porcine nematodes, solely concerning the genus *Oesophagostomum* (Roepstorff et al., 1987; Bjørn et al., 1990; Bauer and Gerwert, 2002; Gerwert et al., 2002; Macrelli et al., 2019; Pettersson et al., 2021a). To date, there is no evidence for AR in other porcine nematode species such as *A. suum*, *Trichuris suis* or *Metastrongylus* spp. This situation is fundamentally different when compared to the severe and widely spread AR problematic in ruminants and horses (Charlier et al., 2022; Nielsen, 2022). However, this could potentially be due to the fact that fewer studies have been carried out on pig nematodes compared to other host animal species (Kaplan et al., 2023). Additionally, the type of farms sampled in this study could have an effect on the chances to detect AR. The farm selection for this study was based on the assumption that farms with outdoor pens or access to pasture are associated with a higher risk for parasite infections compared to conventionally reared animals (Roepstorff and Nansen, 1994; Carstensen et al., 2002). One would generally expect a high risk of resistance development from methods observed on the participating farms, such as use of the active compounds for several years without change or changes within the same class of drugs, the almost exclusive use of BZs, frequent treatment up to every six weeks, or dilution of the dose calculated for one day over several days and the potential accumulation of resistant alleles in the total worm population over time due to lack of effective hygiene e.g. on outdoor runs and pastures. However, at the same time the rearing conditions in alternative pig farms, such as access to pasture, continuous occupancy of pens instead of all-in-all-out systems, non-slatted floors, allow a large number of eggs to remain in the environment. Those eggs constitute a large refugium that escapes treatment for a long period of time in extensive pig farms (Roepstorff and Nansen, 1994). Additionally, sows which were not as intensively treated as fatteners and are the main age group being infected with strongyles (Murrell, 1986; Roepstorff and Jorsal, 1989; Carstensen et al., 2002; Kochanowski et al., 2017; Hernandez et al., 2023) may also contribute to the refugium. This poses the question, if there is a higher risk of AR emergence in intensive indoor systems, where the refugium is potentially smaller due to frequent removal of parasite eggs by disinfection and slatted floors. However, the design of the present study was not suitable to investigate effects of different management practices such as conventional, intensive indoor husbandry versus access to outdoor pens and pastures but this should be investigated in future project phases.

The implementation of the FECRT as detailed by Kaplan et al. (2023) on pig farms was subjected to several challenges and limitations: (i) The lack of individually numbered ear tags made the identification of the animals after a period of two weeks quite challenging and time consuming. Consequently, a substantial number of sampled animals per group was necessary to guarantee an adequate number of paired samples. (ii) Short treatment intervals, of sometimes every six weeks in some fattening herds, which required the willingness of the farmers to suspend their treatment regime for this study. (iii) The lack of standardised weight determination across the various farms. (iv) Individual dosing was not possible and a group treatment approach, based on the heaviest animal in the group, was chosen. For hygienic reasons, especially, because of the prevailing African swine fever virus cases on German pig farms and in wild boars (Sauter-Louis et al., 2021; Authority (EFSA) et al., 2024), and transportation issues it was not possible that the investigator would bring its own scale on the farm. Weight tapes available for farmers in Germany only provided estimates for carcass weight of fatteners. Therefore, the weight of 6 out of 13 animal groups (Table 1, Table 2) was estimated based on the breed, age, size and body condition. With regard to group treatment, it should also be mentioned that it was not possible to check if the animals ingested the correct dosage. Despite the potential misestimation of the weight or low treatment intake resulting in incorrect dosing of the animals, no reduced efficacy of FBZ against strongyles was detected either on farms with or without scales. This suggests that a sufficient dose of FBZ was taken up by the pigs and strongyle communities on all investigated farms were susceptible. Noteworthy, the advised dosage of 5 mg FBZ/kg body weight is 20 times higher than the lowest effective dose against susceptible *O. dentatum* strains (Praslička et al., 1997), which should prevent underdosing in many cases even if weight estimates were imprecise.

Actually, there are to date no guidelines available regarding specific FECRT methods or target efficacies for *A. suum* (Coles et al., 1992; Kaplan et al., 2023). The main reason for the lack of specific guidelines is that ascarid faecal egg counts of pigs are highly variable and potentially confounded by coprophagy (Boes et al., 1997; Roepstorff et al., 1997, 1998; Kaplan et al., 2023) which makes the FECRT interpretation unreliable. The closest related species addressed within the W.A.A.V.P. guideline from 2023 (Kaplan et al., 2023) are the horse parasites *Parascaris* spp. When interpreting the *A. suum* FECRT estimates using the classification criteria outlined by Denwood et al. (2023) and applying the grey zone of 95–99.9 % for the research protocol of *Parascaris* spp., FECR 90 % CrIs based on all EPG data (including egg counts <200 EPG) for ascarid populations on three (F2F, F11P, F11B, F15F), two (F9P, F9F, F13aF), one (F14P) and four (F3P, F5F, F7F, F8F) farms would be classified as resistant, low resistant, inconclusive and susceptible, respectively. Even when setting a target efficacy of 95 %, in order to minimize effects of falsely interpreted FECRT e.g. due to coprophagy occurring in pigs, 25 % of the populations would be interpreted as resistant. Interestingly, the egg counts post-treatment ranged between 1 and 12 with the exception of one animal from F11P (460 EPG). This particular animal exhibited a substantial decline in of strongyle eggs, from over 4000 EPG pre-to 0 EPG post-treatment. Furthermore, upon examination of the apparent resistant ascarid populations on F2F and F11P, it was observed that all individuals tested negative for strongyles post-treatment (F15F did not have strongyle infections pre-treatment). These findings suggest that the uptake of FBZ on the individual animal level was adequate. Boes et al. (1997) showed that the number of false-positive animals in their experiments ranged between 29 and 76 % of all pigs with positive *A. suum* egg counts, and the false-positive egg counts ranged between 20 and 1060 EPG (modified McMaster method with a multiplication factor of 20). When considering low egg counts (<200 EPG) as negative and applying a grey zone of 95–99.9 % for the research protocol of *Parascaris* spp., FECR estimates and 90 % CrIs for ascarid populations on one (F11P), one (F5F), one (F14P) and seven (F3P, F7F, F8F, F9P, F9F, F11B, F13aF, F15F) farms would be classified as resistant, low resistant, inconclusive and susceptible, respectively. With a target efficacy of 95 % only 2 out of 11 populations would be interpreted as resistant (F11P with 90 % CrIs of 65.5–71.2 % and F14P with 16.7–100 %). Overall, excluding egg counts <200 EPG pre- and post-treatment seems to be a promising approach to reduce the possibility of falsely interpreting animal groups as resistant. However, more research with larger sample sizes is needed to confirm this. In some cases (F5F, F11P, F14P) excluding egg counts <200 EPG resulted in decreased FECR 90 % CrIs. Hence, excluding egg counts <200 EPG may not be able to overcome the problematic of false positive animals due to coprophagy completely. It therefore remains difficult to make a final statement on the AR status in *A. suum* based on the FECRT data only.

To overcome the mentioned limitations and to gain more detailed information on the resistance status of the parasite communities, further methods complementing the FECRT are required for both strongyles and ascarids. While for strongyles multiple *in vitro* tests such the EHA and the LDA as well as molecular tests quantifying the presence of BZ-resistance-associated polymorphisms in the isotype-1 β-tubulin gene have been well established in ruminant parasites and are also available for canine, human and equine parasites (Tydén et al., 2014; Avramenko et al., 2019; George et al., 2022; Venkatesan et al., 2023), they have to the best knowledge of the authors never been applied to porcine strongyles. However, the EHA and the LDA were not considered to be included in the design of the present study due to the logistics of sampling and subsequent cooling of the samples, which may potentially interfere with egg/larval development. Therefore, it was decided to establish molecular tests for the presence of BZ-resistance in *Oesophagostomum* spp., the most important strongyle parasite of pigs. In contrast to strongyles, no molecular or *in vitro* tests were available for characterizing populations of *A. suum* but an *in ovo* LDA had been described for other ascarids (Tydén et al., 2016; Tarbiat et al., 2017). Molecular tests were excluded since there is no clear ascarid ortholog to the isotype-1 β-tubulin gene of strongyles (Roose et al., 2021; Özben et al., 2022) and no evidence that BZ-resistance associated isotype-1 β-tubulin sequence polymorphisms exist, was obtained in a worldwide screen (Jones et al., 2024).

Due to the previous reports of BZ AR in *Oesophagostomum* spp. (Bauer and Gerwert, 2002; Gerwert et al., 2002) and to affirm our findings from the FECRT, it was aimed to establish a method to analyse the occurrence of isotype-1 β-tubulin SNPs associated with BZ-resistance in various nematodes (e.g. Avramenko et al., 2019; Dilks et al., 2021; Venkatesan et al., 2023). A deep amplicon sequencing approach using two separate amplicons – amplicon A covering codons 134 and 167, and amplicon B covering codons 198 and 200 – analysed with Illumina 300 bp paired-end read sequencing technology was used to characterise the BZ-resistance status of *Oesophagostomum* spp. communities from participating farms. Corroborating the results from the FECRT, no mutations that potentially cause BZ-resistance were found. However, it was not possible to reliably differentiate between the *Oesophagostomum* spp. due to high similarities in the amplicon A. Potentially, a longer sequence covering all codons of interest could solve this problem. Unfortunately, a large intron (ca. 2000 bp) in the isotype-1 β-tubulin gene did not allow Illumina sequencing, which is restricted to a maximum of approximately 500 bp paired end reads. In the future, other sequencing methods may allow larger amplicons, such as PacBio or Oxford Nanopore Technologies could be evaluated for this purpose and compared with Illumina results. Overall, this was the first time that a deep amplicon sequencing approach was successfully used to describe the isotype-1 β-tubulin regions putatively associated with BZ-resistance in the pig nematodes. The fact that the approach is currently limited to *O. dentatum* and *O. quadrispinulatum*, is only a minor disadvantage since these are the by far most abundant strongyle parasites of pigs and also the only ones for which resistance has ever been reported before (Bauer and Gerwert, 2002; Gerwert et al., 2002).

Interestingly, previous results based on ITS-2 deep amplicon sequencing of pre-treatment samples of the participating farms showed that mixed infections with *O. dentatum* and *O. quadrispinulatum* are common, and even mixed infections with the hookworm *Globocephalus urosubulatus* were found (Fischer et al., 2024). It was also shown that *O. dentatum* was by far the most frequently observed nematode in the mixed species infections representing at least 73.9 % of the DNA reads (Fischer et al., 2024). However, there is a gap of knowledge for target efficacies for other strongyle species or mixed infections in pigs (Kaplan et al., 2023). Hence, the aim was to use ITS-2 metabarcoding to determine effects of BZ treatment on *Oesophagostomum* spp. community composition. When interpreting the nemabiome data it has to be mentioned, that the obtained species frequencies were not corrected for species-specific biases as foreseen in the original protocols for sheep and cattle parasites (Avramenko et al., 2015; Redman et al., 2019). Although a definitive statement is not possible, it seems justified to compare the pre- and post-treatment data, particularly since no relevant differences in PCR efficacy should be expected with the two major parasite species being so closely related regarding their ITS-2 sequences as *O. dentatum* and *O. quadrispinulatum*. The results of the nemabiome analysis based on two post-treatment samples showed that the proportion of *O. quadrispinulatum* increased significantly (p < 0.001) after treatment. This corresponded with the results of Praslička et al. (1997), who observed a higher efficacy of FBZ against *O. dentatum* than against *O. quadrispinulatum.* The underlying cause for the different efficacies in both species is unknown. Praslička et al. (1997) assumed that the distribution of the two species in the large intestine (*O. quadrispinulatum* further proximal, *O. dentatum* distal) and the resulting differences of exposure time, concentration of the drug and its metabolites may be the reason for lower efficacy against *O. quadrispinulatum.* This was also assumed for pyrantel citrate (Bjørn et al., 1989) and for ivermectin (Várady et al., 1996). As shown by Praslička et al. (1997), FBZ was less efficient against “immature worms” in both *Oesophagostomum* species. Therefore, it can be speculated that the increase of *O. quadrispinulatum* after treatment is based on a longer prepatency (18–42 days) compared to *O. dentatum (*18–28 days) and survival of histotropic larval stages followed by maturation to adults in the two weeks post-treatment (Várady et al., 1996). However, as can be assumed from the FECR estimates for both farms (99.8 % and 99.9 %, respectively, exceeding the target efficacy of 99.0 % for *O. dentatum),* the lower BZ efficacies of the less prevalent species *O. quadrispinulatum* does not appear to have a strong impact on the FECRT interpretation for porcine strongyles in our setting.

Until now, the deep amplicon sequencing is cost intensive and requires access to specially equipped labs as well as bioinformatic knowledge in order to interpret the results correctly. However, in the fast developing field of NGS it is conceivable that in future it will be feasible to analyse clinical samples.

In order to overcome the problem of coprophagy-associated false positive *A. suum* egg shedding and in the absence of suitable data to develop molecular tests, an *in vitro* assay (LDA) was implemented to verify FECRT results. Previously published *in vitro* assays that were reported for the equine parasite *Parascaris* spp. (Tydén et al., 2016) and the poultry parasite *A. galli* (Tarbiat et al., 2017) were successfully adapted to the requirements of *A. suum*. The results of the developed *in ovo* LDA showed a range of EC_50_ values between 1.49 and 3.30 μM TBZ. In comparison, the EC_50_ values for *A. galli* and *Parascaris* spp. were 0.5 μM and 55 μM TBZ, respectively (Tydén et al., 2016; Tarbiat et al., 2017). Interestingly, in our results, the farm with the highest EC_50_ value showed full *in vivo* efficacy of the FBZ treatment (F3P, FECR 99.9 %, 90 % CrIs 99.7–100 %). Moreover, the farm with the lowest EC_50_ value was the one with the lowest FECR (F11P, FECR 73 %, 90 % CrIs 70.5–75.2 %)). On this particular farm, only one single piglet out of nine showed a positive egg count post-treatment (460 EPG). Based on the FECR alone (both FECRT calculations with and without egg counts <200 EPG), the farm F11P would be assigned a BZ resistant status. However, the low EC_50_ value shows that the FECR is most likely confounded, e.g. by false positive egg shedding in individual pigs or too low FBZ doses in individual pigs. Overall, the results of the *in ovo* LDA lead to the conclusion that none of the *A. suum* populations analysed was resistant to FBZ. We suggest a provisional EC_50_ of 3.90 μM TBZ (mean EC_50_ + 3 × SD) as cut-off for susceptible populations. This value needs further adaptation in the future when additional *A. suum* populations from different geographic regions were analysed using the *in ovo* LDA. Due to the lack of true BZ-resistant populations *in vivo*, it was not possible to compare the EC_50_ values between resistant and susceptible populations. It is conceivable that the newly established LDA could also be used for the human pathogenic parasite *Ascaris lumbricoides* in the future. Soil-transmitted helminths (STH), including *A. lumbricoides*, are estimated to infect 24 % of the world's populations (www.who.int, last visited June 17, 2024). The World Health Organisation (WHO) currently recommends mass drug administration programs without previous individual diagnosis, which has led to an increasing concern regarding emergence of BZ-resistance (Ásbjörnsdóttir et al., 2017; Krücken et al., 2017). Due to the high genetic similarity (if not identity) between *A. suum* and *A. lumbricoides*, it can be expected that the *in ovo* LDA described in this study will be readily applicable to analyse *A. lumbricoides* strains and to compute EC_50_ values. The LDA is simple to conduct, low-priced and therefore readily suitable for clinical cases. However, the preliminary EC_50_ of 3.90 μM TBZ as cut-off for susceptible strains will need to be further refined in future studies.

It was not aimed to establish a next-generation sequencing approach for *A. suum*, analogue to the deep amplicon sequencing of the isotype-1 β-tubulin gene of strongyles. As stated above, polymorphisms in the isotype-1 β-tubulin gene associated with BZ-resistance in strongyles have not yet been detected in *A. suum*, *A. lumbricoides* or *P. univalens* populations (Krücken et al., 2017; Özben et al., 2022; Jones et al., 2024). Even phenotypic resistant populations of *A. lumbricoides* and *P. univalens* lacked the SNPs in the isotype-1 β-tubulin gene (Krücken et al., 2017; Özben et al., 2022). Additionally, the differences between strongylid and ascarid β-tubulin isotype genes do not allow predictions regarding the isotype involved in BZ-resistance in ascarids (Demeler et al., 2013; Krücken et al., 2017; Roose et al., 2021; Jones et al., 2024). This lack of knowledge about the mode of BZ-resistance in ascarids hinders the mere copying of the methods used for strongyle nematodes. The β-tubulin isotype genes of *A. lumbricoides Alu/asu-bt-A* and *Alu/Asu-bt-B* have been suggested as the most promising isotypes, when looking for molecular markers of BZ-resistance (Roose et al., 2021). However, further investigations, such as (i) the comprehensive application of the *in ovo* LDA to corroborate the preliminary cut-off value for susceptible strains and to reliably identify resistant ascarid strains in pigs or humans, (ii) a more detailed characterisation of the β-tubulin family in ascarids with resistant phenotype, and (iii) NGS approaches like the whole genome sequencing of phenotypic resistant specimens, would be necessary for the detection of potential resistance mechanisms in ascarids.

In conclusion, the most abundant porcine gastrointestinal nematodes *Oesophagostomum* spp. and *A. suum* were found to be susceptible to FBZ on all German farms with outdoor access included in the present study. The FECRT is a suitable tool to analyse porcine strongyle parasite communities, even when mixed infections with both nodular worms are common. Nemabiome analyses provided interesting insight into the composition of pre- and post-treatment strongyle communities. In the future, *Oesophagostomum* spp. communities can be analysed for the presence of resistance-associated SNPs using the isotype-1 β-tubulin gene next generation sequencing approaches established here. In contrast, the FECRT is not sufficiently reliable to analyse *A. suum* populations due to highly variable and false-positive egg counts due to coprophagy. The FECRT, calculated with considering low *A. suum* egg counts (<200 EPG) as negative, is assumed to be more suitable to counteract effects of false-positive egg counts due to coprophagy. It is suspected, that a larger number of tested individuals may be necessary to increase the probability of positive (>200 EPG) egg counts pre-treatment. However, further studies need to be conducted to validate this approach of FECRT calculations for *A. suum* and its suitability to cope with false-positive egg counts. Since the resistance mechanism of ascarids against BZs has not been identified and molecular markers are not yet available, deep amplicon sequencing is not yet an useful approach for diagnostic purposes of BZ-resistance in field studies. Therefore, an *in vitro* test, the *in ovo* LDA, was developed to assess the drug efficacy independently of egg counts. Based on the examination of 13 different susceptible *A. suum* populations, we suggest a preliminary EC_50_ of 3.90 μM TBZ as cut-off for susceptible strains that should be further refined in future studies. The combination of the FECRT and *in ovo* LDA revealed no BZ-resistance in the analysed *A. suum* populations.

## CRediT authorship contribution statement

**Hannah RM. Fischer:** Writing – original draft, Validation, Methodology, Investigation, Formal analysis, Data curation, Conceptualization. **Jürgen Krücken:** Writing – original draft, Visualization, Supervision, Project administration, Methodology, Funding acquisition, Formal analysis, Conceptualization. **Stefan Fiedler:** Writing – review & editing, Methodology, Investigation, Formal analysis. **Stig M. Thamsborg:** Writing – review & editing, Resources. **Hendrik Nienhoff:** Writing – review & editing, Methodology. **Stephan Steuber:** Writing – review & editing, Supervision, Methodology, Conceptualization. **Ricarda Daher:** Writing – review & editing, Supervision, Methodology, Investigation, Funding acquisition, Conceptualization. **Georg von Samson-Himmelstjerna:** Writing – review & editing, Supervision, Project administration, Methodology, Funding acquisition, Conceptualization.

## Availability of data and materials

All data generated or analysed during this study are included in this published article and its supplementary information files with the following exceptions: Raw deep sequencing data of the β-tubulin gene regions were deposited in the short read archive (SRA) of GenBank under the BioProject ID PRJNA1201733. Raw deep sequencing data of the ITS-2 gene region were deposited in the short read archive (SRA) of GenBank under the BioProject ID PRJNA1111032 with the BioSample IDs SAMN41380804, SAMN41380807, SAMN41380812 and SAMN41380813. The Sanger sequenced β-tubulin gene of *Oesophagostoumum* spp. are available under the accession no. PV442195-PV442203. The ITS-2 sequence data for sequence variants of *Oesophagostoumum* spp. post-treatment that were not in GenBank before, were deposited in GenBank under the accession no. PP785341 – PP785343.

## Note:

Nucleotide sequence data reported in this paper are available in the GenBank™ database under the accession numbers PRJNA1201733, PRJNA1111032 (SAMN41380804, SAMN41380807, SAMN41380812 and SAMN41380813), PP785341, PP785342, PP785343, PV442195, PV442196, PV442197, PV442198, PV442199, PV442200, PV442201, PV2202, PV442203.

## Funding

This study was a collaboration between the Freie Universität Berlin and the Federal Office of Consumer Protection and Food Safety (BVL) and funded by the BVL (contract no. 2021000168).

## Conflict of interest

The authors declare the following financial interests/personal relationships which may be considered as potential competing interests: Georg von Samson-Himmelstjerna (GvSH) reports financial support was provided by Federal Office of Consumer Protection and Food Safety, Berlin, Germany. GvSH is a member of the editorial board of Int. J. Parasitol. Drugs Drug Rest. Furthermore, he declares that he has previous and ongoing research and consultancy collaborations with several veterinary pharmaceutical and diagnostic companies. All other authors declare no conflict of interest.

## Appendix A Supplementary data

Multimedia component 4 Multimedia component 4

Multimedia component 5 Codon-based substitution model maximum-likelihood phylogenetic tree computed from codon sequences of the *Oesophagostomum* spp. β-tubulin gene (forward primers Beta_F3, Beta_F1, reverse primer Beta_R; Table S1) and β-tubulin sequences from various nematode species and isotypes. The cluster of isotype-1 β-tubulin isotype-1sequences is highlighted in green. Clade III nematodes refer to *A. suum*, *A. lumbricoides* and *P. univalens*. Branch support is given as SH-aLRT support (%)/ultrafast bootstrap support (%). The scale bare indicates a distance of 0.2 substitutions per site. Multimedia component 5

Multimedia component 6 Multimedia component 6

Multimedia component 7 Expected and observed SNPs frequencies (%). Results show the isotype-1 β-tubulin isotype-1gene region deep amplicon sequencing with artificially generated sequences used as PCR template. Sequences containing SNPs at codon positions 134 and 167 (amplicon A, blue) and at codon positions 198 and 200 (amplicon B, red) where mixed in three different ratios, respectively (susceptible/resistant: 20/80 %, 50/50 % and 80/20 %) and were plotted against the observed SNP frequency by deep amplicon sequencing. The slopes of the linear regressions ranged from 0.9019 to 1.006 and did not differ significantly from 1 (p values: 0.891, 0.945, 0.130 and 0.130, for the regression lines of Q134H, F167Y, E198A and F200Y respectively; GraphPad Prism 5.03). Multimedia component 7
