# Supplementary figures and images for: Faecal egg count reduction test, deep amplicon sequencing of isotype-1 β-tubulin gene and in ovo larval development assay reveal susceptibility to benzimidazoles of porcine nematodes Oesophagostomum spp. and Ascaris suum in outdoor-reared pigs in Germany
Source: Int J Parasitol Drugs Drug Resist. 2025 Aug 28;29:100612. doi: 10.1016/j.ijpddr.2025.100612 (PMC12444184; doi:10.1016/j.ijpddr.2025.100612)

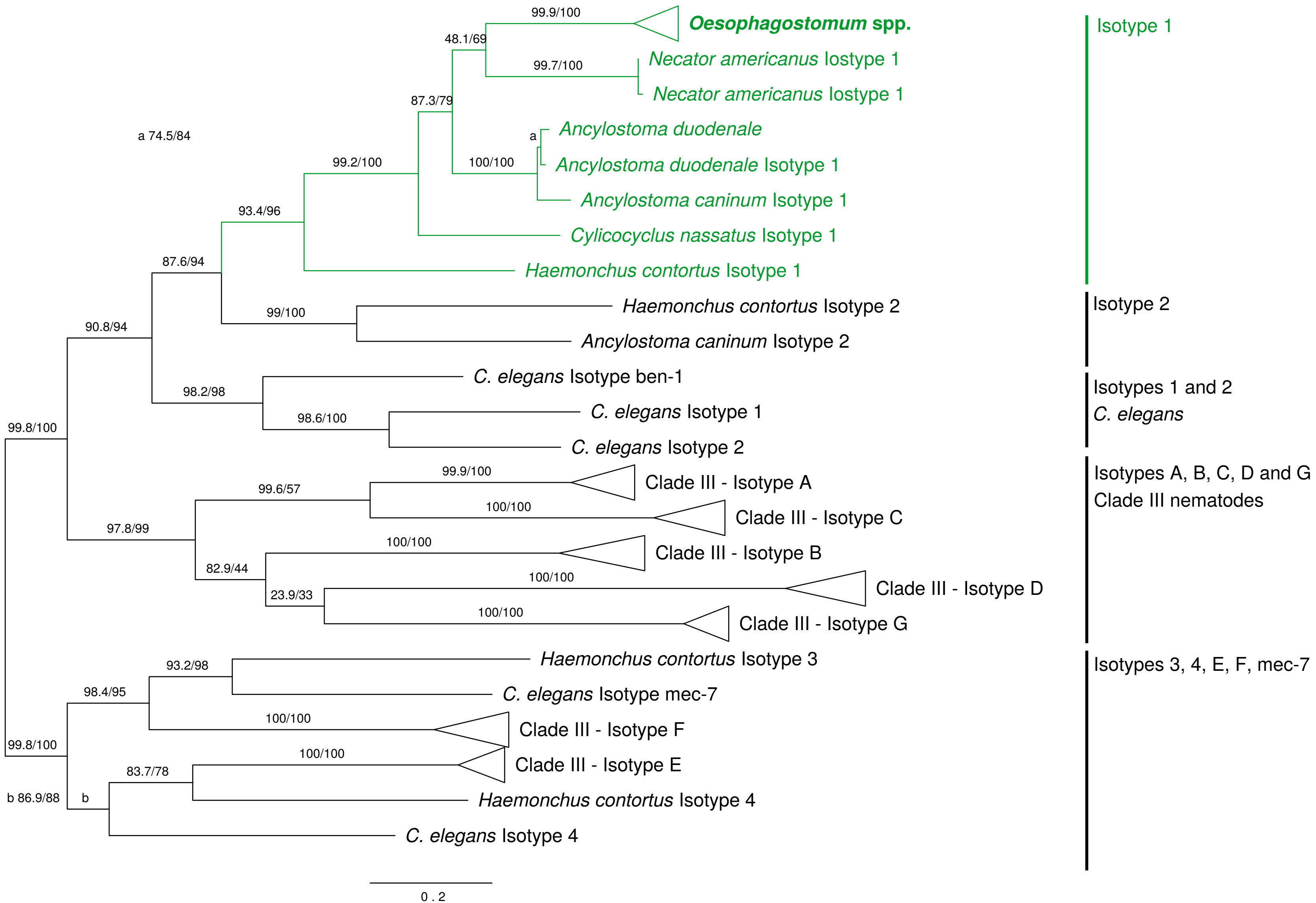

Supplement: Multimedia component 5 — Codon-based substitution model maximum-likelihood phylogenetic tree computed from codon sequences of the Oesophagostomum spp. β-tubulin gene (forward primers Beta_F3, Beta_F1, reverse primer Beta_R; Table S1) and β-tubulin sequences from various nematode species and isotypes. The cluster of isotype-1 β-tubulin isotype-1sequences is highlighted in green. Clade III nematodes refer to A. suum, A. lumbricoides and P. univalens. Branch support is given as SH-aLRT support (%)/ultrafast bootstrap support (%). The scale bare indicates a distance of 0.2 substitutions per site. [file mmc5.pdf]

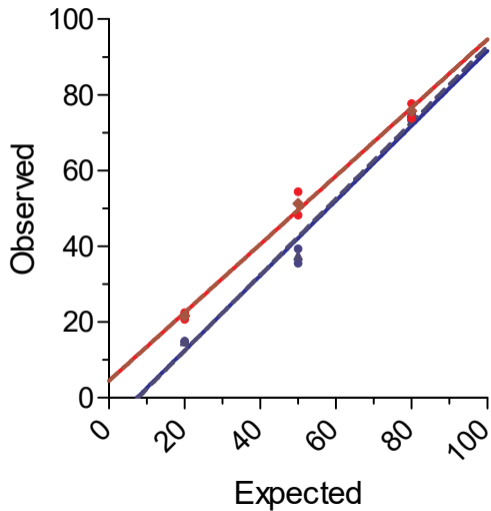

- Q134H:  $y = (-7.325 \pm 4.050) + (0.9894 \pm 0.07274)x$
- F167Y:  $y = (-7.786 \pm 4,239) + (1.006 \pm 0.07613)x$
- E198A:  $y = (4.444 \pm 2.869) + (0.9019 \pm 0.05154)x$
- F200Y:  $y = (4.444 \pm 2.869) + (0.9019 \pm 0.05154)x$

Supplement: Multimedia component 7 — Expected and observed SNPs frequencies (%). Results show the isotype-1 β-tubulin isotype-1gene region deep amplicon sequencing with artificially generated sequences used as PCR template. Sequences containing SNPs at codon positions 134 and 167 (amplicon A, blue) and at codon positions 198 and 200 (amplicon B, red) where mixed in three different ratios, respectively (susceptible/resistant: 20/80 %, 50/50 % and 80/20 %) and were plotted against the observed SNP frequency by deep amplicon sequencing. The slopes of the linear regressions ranged from 0.9019 to 1.006 and did not differ significantly from 1 (p values: 0.891, 0.945, 0.130 and 0.130, for the regression lines of Q134H, F167Y, E198A and F200Y respectively; GraphPad Prism 5.03). [file mmc7.pdf]
