# Supplementary material for: Faecal egg count reduction test, deep amplicon sequencing of isotype-1 β-tubulin gene and in ovo larval development assay reveal susceptibility to benzimidazoles of porcine nematodes Oesophagostomum spp. and Ascaris suum in outdoor-reared pigs in Germany
Source: Int J Parasitol Drugs Drug Resist. 2025 Aug 28;29:100612. doi: 10.1016/j.ijpddr.2025.100612 (PMC12444184; doi:10.1016/j.ijpddr.2025.100612)
Supplement: Multimedia component 6 [file mmc6.pdf]

Table S6 – Filtering statistics of the raw reads

sample	raw	filtered	denoised_fwd	denoised_rev	merged	no_chim
2.1-AmpliconA	7688	6831	6763	6757	6657	6657
2.1-AmpliconA-post	260	231	198	197	159	159
2.2-AmpliconA	16200	14338	14277	14253	13900	13584
3-AmpliconA	12244	10704	10699	10679	10415	10415
5-AmpliconA	7543	6731	6538	6488	6304	6304
6-AmpliconA	10641	9342	9171	9130	8882	8882
7-AmpliconA	119	108	102	101	44	44
8-AmpliconA	7995	7019	6856	6815	6692	6692
10-AmpliconA	8839	7708	7683	7702	7460	7460
10-AmpliconA-post	10529	9077	8962	8959	8711	8711
11-AmpliconA	11953	10857	10831	10828	10458	9579
13-AmpliconA	232	215	204	201	174	174
14-AmpliconA	13482	11705	11639	11637	11336	10793
Od1-AmpliconA	11054	9584	9579	9579	9553	9553
Od2-AmpliconA	11458	10168	10168	10095	10072	10072
Od3-AmpliconA	15056	13183	13182	13176	13157	13157
Oq1-AmpliconA	7846	7040	7040	7039	7030	7030
Oq2-AmpliconA	789	710	708	708	705	705
Oq3-AmpliconA	13022	11558	11553	11555	11536	11354
2.1-AmpliconB	14042	13582	13528	13543	13161	12597
2.1-AmpliconB-post	13889	12198	12067	11932	11067	10437
2.2-AmpliconB	31634	30599	30496	30541	29523	26987
3-AmpliconB	23829	23086	23065	22966	22278	20908
5-AmpliconB	22702	21551	21484	21484	21416	21295
6-AmpliconB	23023	21827	21806	21802	21776	21714
7-AmpliconB	264	257	246	228	208	208
8-AmpliconB	20409	19501	19414	19361	18999	18938
10-AmpliconB	18481	17892	17823	17824	17189	15489
10-AmpliconB-post	19807	19108	19070	19068	18645	18524
11-AmpliconB	24300	23734	23673	23675	22740	20712
13-AmpliconB	14856	13622	13543	13527	12692	12692
14-AmpliconB	26908	26003	25969	25889	24978	22992
Od1-AmpliconB	19135	18610	18609	18608	18376	18376
Od2-AmpliconB	17170	16747	16746	16743	16259	16259
Od3-AmpliconB	27800	27058	27055	27052	26709	26709
Oq1-AmpliconB	13403	13106	13104	13104	12926	12926
Oq2-AmpliconB	1269	1238	1237	1238	1207	1207
Oq3-AmpliconB	25694	25043	25039	25037	24648	24648
2.1-ITS-2-post	71301	54850	54608	54499	54328	53547
10-ITS-2-post	36348	28151	28114	28118	27979	26463
